# Meeting the oral health needs of 12-year-olds in China: human resources for oral health

**DOI:** 10.1186/s12889-017-4384-7

**Published:** 2017-06-20

**Authors:** Xiangyu Sun, Eduardo Bernabé, Xuenan Liu, Shuguo Zheng, Jennifer E. Gallagher

**Affiliations:** 1 Department of Preventive Dentistry, Peking University School and Hospital of Stomatology, National Engineering Laboratory for Digital and Material Technology of Stomatology, Beijing Key Laboratory of Digital Stomatology, 22 Zhongguancun Avenue South, Haidian District, Beijing, 100081 People’s Republic of China; 2 King’s College London Dental Institute at Guy’s, King’s College and St Thomas’ Hospitals, Population and Patient Health Division, London, SE5 9RS UK

**Keywords:** Caries risk assessment, Schoolchildren, Parental education, Dental behaviours, Preventive measures, Human resources for oral health, Dental professionals

## Abstract

**Background:**

An appropriate level of human resources for oral health [HROH] is required to meet the oral health needs of population, and enable maximum improvement in health outcomes. The aim of this study was to estimate the required HROH to meet the oral health needs of the World Health Organization [WHO] reference group of 12-year-olds in China and consider the implications for education, practice, policy and HROH nationally.

**Methods:**

We estimated the need of HROH to meet the needs of 12-year-olds based on secondary analysis of the epidemiological and questionnaire data from the 3rd Chinese National Oral Health Survey, including caries experience and periodontal factors (calculus), dentally-related behaviour (frequency of toothbrushing and sugar intake), and social factors (parental education). Children’s risk for dental caries was classified in four levels from low (level 1) to high (level 4). We built maximum and minimum intervention models of dental care for each risk level, informed by contemporary evidence-based practice. The needs-led HROH model we used in the present study incorporated need for treatment and risk-based prevention using timings verified by experts in China. These findings were used to estimate HROH for the survey sample, extrapolated to 12-year-olds nationally and the total population, taking account of urban and rural coverage, based on different levels of clinical commitment (60-90%).

**Results:**

We found that between 40,139 and 51,906 dental professionals were required to deliver care for 12-year-olds nationally based on 80% clinical commitment. We demonstrated that the majority of need for HROH was in the rural population (72.5%). Over 93% of HROH time was dedicated to prevention within the model. Extrapolating the results to the total population, the estimate for HROH nationally was 3.16–4.09 million to achieve national coverage; however, current HROH are only able to serve an estimated 5% of the population with minimum intervention based on a HROH spending 90% of their time in providing clinical care.

**Conclusions:**

The findings highlight the gap between dental workforce needs and workforce capacity in China. Significant implications for health policy and human resources for oral health in this country with a developing health system are discussed including the need for public health action.

**Electronic supplementary material:**

The online version of this article (doi:10.1186/s12889-017-4384-7) contains supplementary material, which is available to authorized users.

## Background

A global strategy for Human Resources for Health [HRH] 2030 was recently approved at the 69th assembly of the World Health Organisation [WHO] [1]. This strategy supports the concept of Universal Health Coverage [2, 3], and the United Nations Sustainable Development Goals for 2030 [4], by ensuring equitable access to health workers within strengthened health systems. The HRH strategy has four goals: First, to “optimise performance, quality and impact of the health workforce through evidence-informed policies on human resources for health, contributing to healthy lives and well-being, effective universal health coverage, resilience and health security at all levels”. Second, to “align investment in human resources for health with the current and future needs of the population taking account of labour market dynamics, to enable maximum improvements in health outcomes, employment creation and economic growth”. Third, to “build the capacity of institutions at sub-national, national and international levels for effective leadership and governance of actions on human resources for health”. Fourth, to “strengthen data on human resources for health, for monitoring of and ensuring accountability for the implementation of both national strategies and the Global Strategy” [1]. The global HRH strategy [1], provides a basis for reviewing and strengthening human resources for oral health [HROH] and health systems between, and within, countries.

Inequalities in oral health workforce distribution are evident globally [5, 6], with almost all developed countries reporting insufficient dentists and specialists, and limited rural coverage [6, 7]. Moreover, all countries are facing a growing and ageing population [8]. Hence, the recommendation of the World Dental Federation [FDI] for optimised health professional workforce planning to meet the increasing need and demand for oral healthcare [9]. In China, though the number of oral health professionals is growing rapidly, demand for care remains rather low, thus unmet need is a major problem, together with evidence of out-of-pocket payments and catastrophic expenditure for oral health care [10, 11].

Whilst much oral disease requires restoration, replacement and repair, in addition to surgical management, there is an important paradigm shift towards prevention [12]. Contemporary guidelines advocate risk assessment [13], and comprehensive management of risk including self-care (toothbrushing, regular use of fluoride, and dietary adjustments to reduce sugar) [13–17], professional care (regular check-ups, topical fluoride application and pit-and-fissure sealants). As 12-year-olds are the reference age for child oral health globally [18, 19], they provide a key group on which to consider oral health planning for HROH from which population extrapolations may be made.

The burden of oral disease is high globally [20], and in China [10, 21]. Furthermore, people with the highest needs may experience challenges in accessing care [10]. HROH in China are evolving and growing as the country goes through a rapid period of transformation. In addition to dentists, which form the basis of HROH, the government has recognised “assistant dentists” who hold lower qualifications but are nonetheless able to perform most dental treatments, including prevention; they achieve this by working under supervision of qualified dentists, or with official permission from local authorities in some rural and mountainous areas, independently. National data suggest that there were only 102.7 dental professionals (dentists and assistant dentists) per million population nationally in 2014 [22], and evidence from northern China also shows that the supply of dental workforce cannot satisfy the demand [23]. The dental workforce requirements to meet the needs of the Chinese population and methods for optimising distribution of HROH remain unexplored.

Methodologies, which address health needs, are important to estimate oral health workforce requirements. A recent review of the literature advocates the importance of needs-led approaches to workforce planning [24], as does the 2030 strategy [1]. Needs-led approaches, whereby the population and epidemiological evidence of their health needs form the basis of the model, have been widely accepted as important [1, 24], and are increasingly successfully being applied in dental workforce planning within the United Kingdom [UK] [25–28]. In these methodologies, needs inform the level of oral care required and then the time necessary to treat each of these conditions is estimated (based on wider research) and the level of HROH required to perform those tasks is derived. They have advantages when compared with some other approaches, as they allow for a fine-grained analysis of the requirements of each medical specialty, independent of the current service utilization ratios, and yet are easy to understand [24].

All countries globally are challenged to consider the provision of universal access to care [2, 3], and their human resource needs within healthcare. Thus, with the available dataset of the 3rd national oral health survey, there is a great opportunity to examine the requirements for workforce capacity development to meet the oral health needs of the world’s largest population, China.

The aims of this study were twofold. First, to estimate the requirement for human resources for oral health to meet the treatment and prevention needs of the WHO reference group of 12-year-olds in China, to enable maximum improvement in health outcomes, based on contemporary approaches to dental care. Second, to extrapolate the findings to consider the workforce needs nationally; and therefore, to enable comparison with the current workforce structure in this country and discussion on the implications for education, practice and policy in relation to oral health.

## Methods

### Data source

This study used cross-sectional data from the 3^rd^ National Oral Health Survey of China (2005) [29], which covered the four WHO index ages [18]. All 31 provinces of Mainland China participated in the survey, except for Tibet. Participants were selected using multistage stratified cluster sampling. First, each province was divided into urban and rural areas; the former were classified into three strata by population size, whereas the latter were classified into three strata by Gross Domestic Product [GDP]. Second, one city in urban areas and one county in rural areas were randomly selected from each stratum. Hence, three cities from urban and three counties from rural areas were selected from each province. Third, at each school in these cities or counties, a random sample of 20 children aged 12 years was selected from the full name list of students. A target sample of 720 participants was initially set per province, to achieve a national sample of 21,600 12-year-old schoolchildren. Similar approaches were used to achieve coverage of 5-year-olds and adults [11, 30]. The contents of this National Oral Health Survey included two major parts for participants: dental clinical examinations and questionnaire surveys.

Dental clinical examinations were conducted in a standardised manner with participants seated on a chair, and examined using artificial light, plane mouth mirrors and standard WHO CPI probes. All deciduous and permanent teeth were examined and dental caries was diagnosed according to the WHO criteria [18]. Dental calculus was recorded for each tooth where present. Unified training sessions were provided to over 200 survey examiners in Kunming city, Yunnan, before the national survey began. Five percent of participants were re-examined nationally to calculate inter-examiner reliability, and the Kappa score was 0.92. For each participant, dental caries experience was calculated as the total number of decayed, missing and filled permanent teeth [DMFT]. Children’s sex and place of residence (urban or rural) were also recorded whilst their ethnicity was self-assigned using a list of officially recognised ethnic groups in China, with responses later categorised as ‘Han’ or ‘minority ethnic group’.

A self-complete questionnaire survey was used according to the WHO recommendations for oral health surveys [18], with minor modifications in language expression to make it culturally appropriate for China. Dental behavioural and social risk factors were investigated by questionnaire. The former included toothbrushing frequency and frequency of sugar intake. Children reported their toothbrushing frequency ‘less often than daily’, ‘once a day’, and ‘twice a day or more often’; the first two categories were combined into daily or less, and twice a day or more. Children’s sugar intake frequency refers to five common sugary items (biscuits, cake or sweet bread, candy or chocolate, sugared water, soft drinks and fruit juice) on a 6-point ordinal scale. Frequency of ingestion of each sugary item was scored as follows: twice or more a day (2), once a day (1), 2–6 times a week (2/7 = 0.286), once a week (1/7 = 0.143), 1–3 times per month (1/30 = 0.033), seldom/never (0). Weighted scores were chosen to match the lower consumption frequency in each response category. A total score, ranging from 0 to 10, was generated by aggregating scores for the five sugary items. Based on this score, participants were placed in one of two categories: less often than daily *or* once a day or more. Toothbrushing twice a day or more often and sugar intake less often than daily were regarded as positive behaviours, whilst their opposite behaviours were regarded as negative. Paternal and maternal education levels were reported by the children, and regrouped as poor education (if both parents only had compulsory education up to junior middle school), and higher education (if either parent had education of senior middle school or higher).

During the course of the survey, a total of 23,508 12-year-old children was clinically examined, with questionnaire data available for 12,392, as only half of the survey participants were invited to complete questionnaires. Overall, following statistical analyses, post-stratification weights were used to adjust for differences in the age-by-sex-by-ethnicity-by-province distribution between the sample and the general population in the 30 provinces involved in the study, according to the 5^th^ National Demographic Census in 2000 [31]. Analyses also took into account the complex survey design (stratification and clustering) to produce corrected standard errors. All analyses used SPSS 23 (IBM Corporation, Armonk, NY).

### Risk assessment of dental caries

Risk assessment for dental caries involves consideration of the following factors: dental caries experience, behavioural, demographic, geographic and socio-economic factors [13, 14]. We referred to contemporary methodologies of risk assessment for dental caries [13–15, 32], by the American Dental Association [ADA], the American Academy of Paediatric Dentistry [AAPD], the Public Health England [PHE] and the UK National Institute for Health and Care Excellence [NICE]. Based on the above evidence we integrated their recommendations into a novel risk classification method utilising the range of information provided by the questionnaire of 12-year-old sample of the 3^rd^ National Oral Health Survey in China.

In our risk classification method, we considered those who had caries experience (DMFT > 0) as being at *high risk*. At the other end of the spectrum, those who had no caries experience, no negative behaviours and higher parental education were assigned to the *low risk* group. Others, with no caries experience, were divided into two categories as follows: *relatively high risk* (those who had two negative behaviours or those who had one negative behaviour and with poor parental education) and *relatively low risk* (those who had no negative behaviours but with poor parental education or those who had one negative behaviour and with higher education). All cases were thus classified into one of four risk levels. The proportion of each caries risk ‘level’ in the whole, urban and rural population was then calculated (Table 1). As our study sample only included cases who had no missing values in all relevant variables, the impact of missing data was evaluated, comparing the characteristics of participants with complete data (study sample) and those excluded due to missing values in relevant variables, with the Chi-square test.

**Table 1:** Assessment of caries risk levels

Risk level		Caries experience	Dental behaviours	Parental education	Proportion		
					Overall	Urban	Rural
1	Low	DMFT = 0	Both positive behaviours +	Higher education^a^	3.9%	9.6%	1.6%
2	Relatively low	DMFT = 0	No negative behaviours +	Poor education^b^	17.6%	27.5%	13.6%
			OR				
			1 negative behaviour +	Higher education^a^			
3	Relatively low	DMFT = 0	2 negative behaviours +	Any education^c^	52.1%	34.9%	59.1%
			OR				
			1 negative behaviour +	Poor education^b^			
4	High	DMFT > =1	any behaviours + any education^a^		26.4%	28.0%	25.7%

^a^Higher education, those who had at least one parent with senior middle school or higher education

^b^Poor education, those whose parents both had only compulsory education up to junior middle school

^c^Any education refers to either of them

### Risk-based intervention models

We estimated dental care needs in two intervention models based on the above risk assessment, representing maximum (*maxPIM*) and minimum (*minPIM*) professional intervention models. Using this approach, we were able to obtain a range of possible timings needed and associated HROH, which might be more helpful for debate and enables comparison with the current state of play.

In the *maximum professional intervention* model (*maxPIM*), individuals’ frequency of regular check-ups, including professional advice and fluoride varnish application, were assigned in relation to number of visits per year as follows: low risk (×1), relatively low (×2), relatively high (×3) and high (×4). Fissure sealants were provided for all categories except for those designated as low risk. Tooth restorations (or extractions) were only required in the highest risk group. Scaling and polishing were provided for those who had calculus present regardless of caries risk level; the proportion of participants with calculus showed no significant difference when compared among the four caries risk levels using Chi-square test (*p* > 0.05),

In the *minimum professional intervention* (*minPIM*) model, frequency of regular check-ups, including professional advice and fluoride varnish application, was twice a year for high-risk children and once a year for the three other categories. Other aspects of care, such as fissure sealants, tooth restoration, and scaling and polishing were equivalent to the maximum intervention model.

### Estimate of timings of dental care

**Fig. 1:**
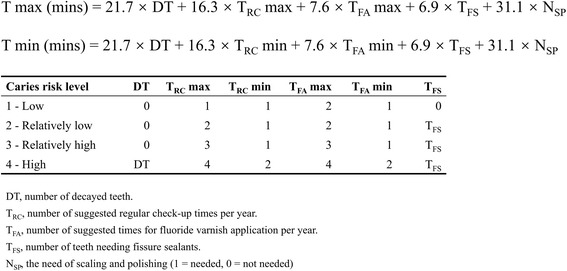
The formula of estimation of timings needed (T max and T min)

Estimated timings of dental care per year of each participant were calculated by aggregating the timing for each item of care based on each individual’s caries risk level. As this modelling involved a conservative approach, dental caries management involved fillings rather than extractions; however, published literature on the timing of procedures suggests that extractions require a similar professional time [33, 34], thus in the models were providing for a conservative approach but recognising there is little impact on time required should a more surgical approach be required. Timings for each measure involved were estimated by consulting nine expert dentists from Peking University School and Hospital of Stomatology and the mean timings were used to obtain a representative data for timings needed for dental professionals in China (published timings in the UK by Bearne and Kravitz [33], and Wanyonyi et al. [34] are shown in Additional file 1: Table S1). The formula for calculating timings required for each participant is presented in Fig. 1.

Total timings for all 12-year-olds nationally (ΣT max and ΣT min) were then estimated by multiplying the sum of timings above with the population-weighting ratio, which was calculated by dividing 12-year-old Chinese population size by the corresponding sample size used for weight.

### Estimate of dental workforce needs

**Fig. 2:**
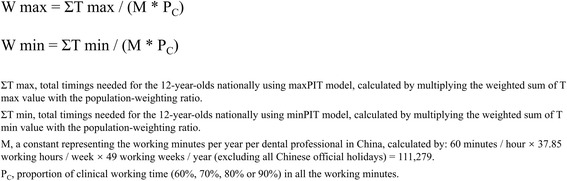
The formula of estimation of workforce needed (W max and W min)

For the next step of estimating dental workforce requirements, dental workforce needs were estimated by dividing total timings needed nationally by the clinical working minutes per year per dental professional. We used data from previous studies on dental professionals in China to compute their working hours per week as 37.85 on average [35]. In the next step, as the proportion of clinical working hours amongst all duties of one dental professional was not known, we were using 80% as the main threshold, whilst 60%, 70% and 90% were set for referring thresholds according to the recommendations obtained after consulting with dental experts in China. Hence, we calculated dental workforce needs per year for 12-year-olds via the formula shown in Fig. 2.

Given that HROH care for all age-groups in the population, we also extrapolated the findings to estimate the gross dental workforce needs for China based on the findings of Wanyonyi et al. [34], which suggest that 1.27% of total care is provided for 12-year-olds.

Thus, for each risk level, the average and total workforce timings required for the weighted sample and total population of 12-year-olds were calculated and extrapolated to estimate the total workforce needs for the whole population. Differences between urban and rural areas and service uptake rate were also taken into account and HROH needs were estimated under different conditions. The analyses were undertaken for both maximum and minimum professional interventions requirements.

## Results

A total of 10,788 children with complete data across all relevant variables were included in this analysis (representing 87% of those who were invited to complete the questionnaire). The mean DMFT was 0.49 (95% CI: 0.45–0.52) and 26.4% (24.6–28.1%) of children had experienced dental caries. The mean number of decayed teeth was 0.44 (0.41–0.47), representing the majority of disease (90%) of the DMFT index. The proportion of children reporting brushing teeth daily or less often, having sugar intake daily or more often, and with poor parental education was 77.2% (75.5–78.8%), 44.2% (42.5–45.8%) and 64.6% (62.7–66.5%), respectively. Just over one fifth of 12-year-old children were at low (3.9%), or relatively low (17.6%) risk, with just over half relatively high (52.1%) and just over one quarter at high (26.4%) risk. There were significant differences between urban and rural areas with the majority of the rural population at *relatively high* risk whilst urban children were more likely to be at low/relatively low or very high risk (Table 1).

Both maximum and minimum professional intervention models are presented in Table 2. The characteristics of the study sample by place of residence are shown in Table 3. Children of the Han (majority) ethnic group and those who have no caries experience were more likely to be included in the study sample. No other differences, even for the key classifying factors, were found between the study sample and those excluded because of missing data.

**Table 2:** Intervention models by caries risk level

	Mean timings needed (in minutes)	Caries risk level			
		1 - Low	2 - Relatively low	3 - Relatively high	4 - High
Maximum professional intervention model					
Tooth restorations	21.7 per tooth	- ^e^	- ^e^	- ^e^	Required^a^
Regular check-ups	16.3 each time	Once a year^c^	Twice a year^a,c^	3 times a year^c^	4 times a year^a,c^
Fluoride varnish application	7.6 each time	Twice a year^b^	Twice a year^a,b^	3 times a year^b^	4 times a year^a^
Fissure sealants	6.9 per tooth	- ^b^	Required^a,b^	Required^a,b^	Required^a,b^
Minimum professional intervention model					
Tooth restorations	21.7 per tooth	- ^e^	- ^e^	- ^e^	Required^a^
Regular check-ups	16.3 each time	Once a year^c,d^	Once a year^b,c,d^	Once a year^b,c,d^	Twice a year^c^
Fluoride varnish application	7.6 each time	Once a year^d^	Once a year^d^	Once a year^d^	Twice a year^b^
Fissure sealants	6.9 per tooth	- ^b^	Required^a,b^	Required^a,b^	Required^a,b^

^a^According to AAPD recommendations published in 2013 and ADA recommendations published in 2014

^b^According to PHE recommendations published in 2014

^c^According to NICE recommendations published in 2004

^d^This is set as the bottom line of the measure

^e^This is due to they have no tooth decay for treatment

**Table 3:** Characteristics of the sample of 12-year-olds by place of residence in China (*n* = 10,788)

Factors		Place of residence		Overall (%)^a^
		Urban (%)^a^	Rural (%)^a^	
Sex	Male	53.5%	52.2%	52.6%
	Female	46.5%	47.8%	47.4%
Ethnicity	Han	94.1%	90.4%	91.5%
	Minority ethnic groups	5.9%	9.6%	8.5%
Caries experience	DMFT > 0	28.0%	25.7%	26.4%
	No caries experience	72.0%	74.3%	73.6%
Calculus	No calculus	44.8%	33.5%	36.7%
	One or more teeth with calculus	55.2%	66.5%	63.3%
Parental education	Poor education	36.9%	75.8%	64.6%
	Higher education	63.1%	24.2%	35.4%
Toothbrushing frequency	Daily or less	59.9%	84.2%	77.2%
	Twice a day or more	40.1%	15.8%	22.8%
Sugar intake frequency	Less often than daily	49.1%	58.5%	55.8%
	Daily or more	50.9%	41.5%	44.2%

^a^All percentages shown here are weighted

Based on dental professionals' clinical working hours for management of dental caries and periodontal diseases, average timings of care for the weighted sample and all 12-year-olds nationally were calculated as shown in Table 4. Prevention accounted for the majority of care within the model (93.3-94.8%).

**Table 4:** Mean and total timings for risk-based contemporary dental care amongst 12-year-olds (weighted sample and population) and estimated workforce required based on dental professionals’ clinical working hours for management of dental caries and periodontal health: minimum - maximum professional intervention

		Caries risk level				Total
		1 - Low	2 - Relatively low	3 - Relatively high	4 - High	
Total						
Sample	Mean time per child (minutes)	42–49	134–158	132–180	187–235	143.3–185.3
	Total time for survey sample (minutes)	17,623–20,833	254,206–299,647	743,766–1,012,836	531,910–667,857	1,547,505–2,001,173
Nationally	Total time for all 12-year-olds (minutes)	40,693,369–48,105,399	586,975,926–691,901,533	1,717,397,892–2,338,697,002	1,228,210,547–1,542,120,447	3,573,277,732–4,620,824,383
	Workforce required (60% clinical^a^)	609–720	8791–10,363	25,722–35,028	18,395–23,097	53,518–69,208
	Workforce required (70% clinical^a^)	522–618	7535–8882	22,048–30,024	15,767–19,797	45,873–59,321
	Workforce required (80% clinical^a^)	457–540	6594–7772	19,292–26,271	13,797–17,323	40,139–51,906
	Workforce required (90% clinical^a^)	406–480	5861–6909	17,148–23,352	12,264–15,398	35,679–46,139
Urban						
Sample	Mean time per child (minutes)	41–48	133–157	133–181	183–231	138.1–175.5
	Total time for survey sample (minutes)	12,053–14,316	113,662–134,045	143,941–195,730	159,077–200,706	428,733–544,797
Nationally	Total time for all 12-year-olds (minutes)	27,830,319–33,057,126	262,452,860–309,517,577	332,368,991–451,952,076	367,317,225–463,441,654	989,969,393–1,257,968,433
	Workforce required (60% clinical^a^)	417–495	3931–4636	4978–6769	5501–6941	14,827–18,841
	Workforce required (70% clinical^a^)	357–424	3369–3974	4267–5802	4716–5950	12,709–16,149
	Workforce required (80% clinical^a^)	313–371	2948–3477	3734–5077	4126–5206	11,120–14,131
	Workforce required (90% clinical^a^)	278–330	2621–3091	3319–4513	3668–4627	9885–12,561
Rural						
Sample	Mean time per child (minutes)	45–52	134–158	132–180	189–237	145.5–189.3
	Total time for survey sample (minutes)	5571–6517	140,544–165,602	599,824–817,106	372,833–467,151	1,118,772–1,456,376
Nationally	Total time for all 12-year-olds (minutes)	12,863,050–15,048,273	324,523,066–382,383,956	1,385,028,901–1,886,744,926	860,893,322–1,078,678,793	2,583,308,339–3,362,855,950
	Workforce required (60% clinical^a^)	193–225	4861–5727	20,744–28,259	12,894–16,156	38,691–50,367
	Workforce required (70% clinical^a^)	165–193	4166–4909	17,781–24,222	11,052–13,848	33,164–43,172
	Workforce required (80% clinical^a^)	144–169	3645–4295	15,558–21,194	9670–12,117	29,018–37,775
	Workforce required (90% clinical^a^)	128–150	3240–3818	13,829–18,839	8596–10,771	25,794–33,578

^a^60%/70%/80%/90% clinical: Clinical working hours accounted for 60%/70%/80%/90% of the whole time equivalent clinical working hours of dental professionals based on 37.85 working hours per week and 49 working weeks per year

**Fig. 3:**
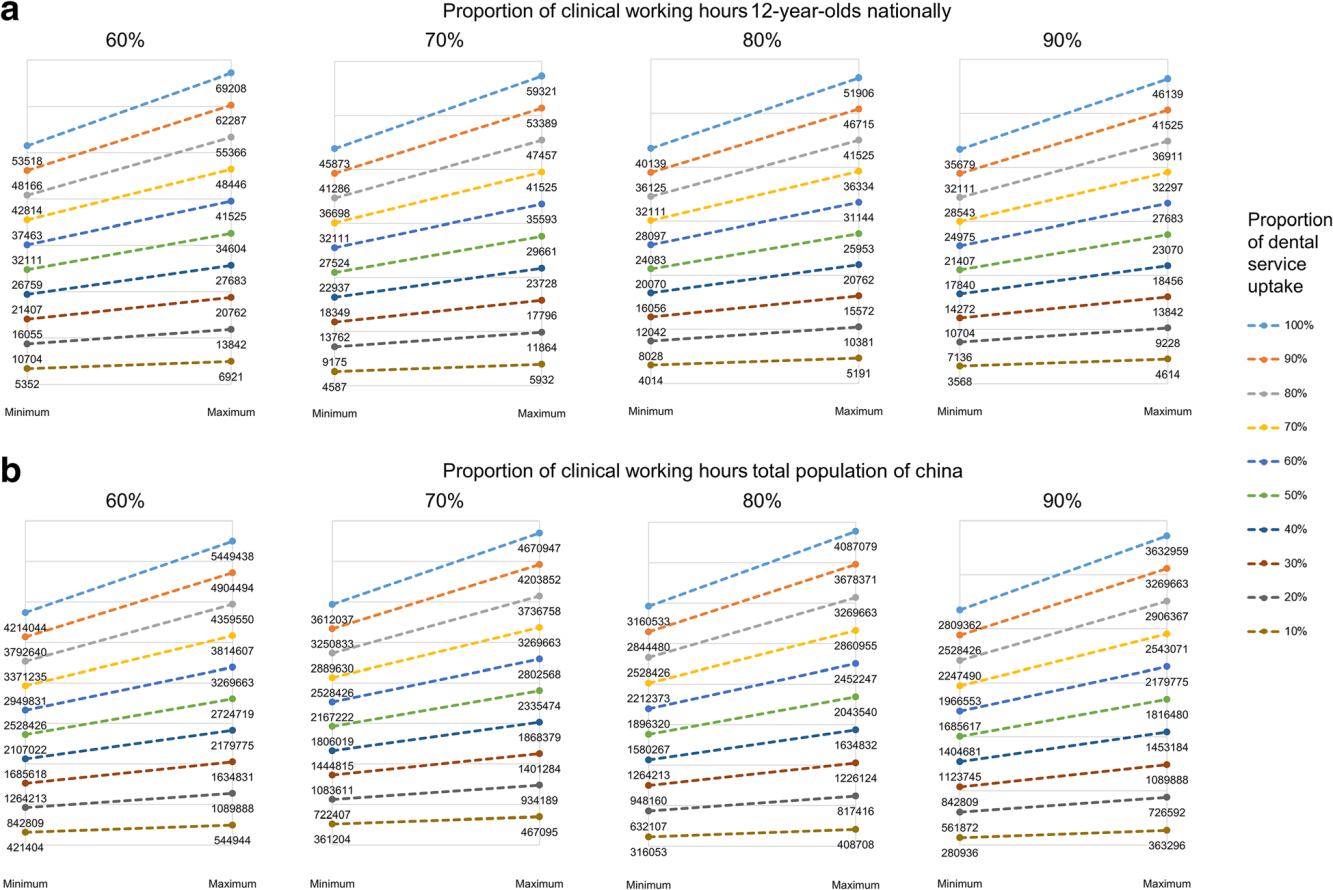
Range of workforce requirements for China based on clinical working hours of dental professionals and service uptake: (**a**) 12-year-olds and (**b**) total population

Nationally, between 3573 and 4621 million minutes of dental professional time were identified as being required to meet 12-year-olds’ treatment and prevention needs. Based on dental professionals spending 80% of their working time delivering clinical care, an estimated 40,139 (*minPIM*) to 51,906 (*maxPIM*) dental professionals are therefore required to deliver contemporary treatment and prevention measures for all 12-year-olds (Table 4 and Fig. 3a).

When the workforce requirements are extrapolated to the total population of China the model suggests a need for 3.16 to 4.08 million dental professionals (Fig. 3b). Most of the workforce requirements are to serve the rural population (72.5%), with just over one quarter (27.5%) required in urban areas.

The above findings are based on the premise that all children receive dental care, whereas service uptake is much lower in reality. Therefore, we consider different levels of dental attendance in our present study to make the estimated condition closer to reality (Fig. 3a and b). However, even if the threshold of clinical working hours increases to 90%, and only 10% of the population take up all treatment and preventive measures, even then approximately 280,936 (*minPIT*) to 363,296 (*maxPIT*) dental professionals are required for the whole population (Fig. 3b).

## Discussion

This study shows the disparity between professional time required to meet population needs, ensure fair access to care, and maximise health outcomes for 12-year-olds in China. We found a large gap between current dental workforce numbers and estimated workforce needs in China, even with the minimum professional intervention model. HROH data suggest that there were only 57,828 dental professionals (dentists and assistant dentists) registered in China in 2005 [22], and although registrants nationally increased to 140,454 in 2014 [36], capacity is low and access limited; thus, unmet treatment and prevention needs remain a major problem for the Chinese population. Based on our minimum professional intervention modelling, even if dental professionals commit 90% of their working hours to clinical treatment and prevention, this is estimated to just serve approximately 5% of the population (Fig. 3b). Our model suggest that the oral health needs of the 12-year-old total population would utilise 25-35% of current actual HROH in China, even if dental professionals endeavour to spend 90% of their working hours in providing direct clinical care; therefore, urgent action is required. Of course not everyone accesses dental care, either because of personal choice or higher level factors which act as barriers [37]. However, as the population of China increases it is very clear that HROH capacity is inadequate and even more so if dental professionals limit their clinical working hours to less than full time.

We also found clear distinctions between urban and rural areas, indicating that the rural population need more dental professionals to cover their treatment and prevention requirements and thus ensure fair access. Given that a considerable proportion of HROH in China are working in urban areas [7, 10, 23], it will be even more difficult to meet the justified needs of rural people. This forced us to reflect on how the oral health care system in China may be improved to ensure sufficient HROH, and coverage, to meet the need and demands of the population, with the latter predicted to grow in the coming years in parallel with rapid economic development in this country.

Based on the national survey findings, contemporary dental care needs of 12-year-olds involve preventive measures which may be delivered by dental hygienists rather than dentists [38]. Additionally, routine restorative dental care, as well as prevention, may be delivered by dental therapists [38–40]. In light of these considerations, future planning should consider how effective and efficient care may be delivered in rural settings using enhanced workforce skill mix and possibly involving schools rather than merely developing expensive dental clinics. It is a real opportunity to establish a contemporary workforce espousing preventive philosophy, and support families who may not be able to access traditional health settings.

These developments should be part of wider initiatives to promote health in general and introduce universal health coverage. To that end, we highlight the importance of promoting health in line with the *Shanghai Declaration on Promoting Health in the 2030 Agenda for Sustainable Development* [41], *and* the *Ottawa Charter for Health Promotion* [42, 43]. The latter includes five areas for action: building healthy public policy; creating supportive environments; strengthening community action; developing personal skills; and, reorientating health services towards prevention. First, in relation to healthy public policy, it is recommended that policy makers should control sugar production, import and sales [13, 15–17], as it is a key risk factor for dental caries and other non-communicable diseases. Additionally, there needs to be policy to develop and shape the future dental workforce. Second, to ensure supportive environments, there should be reduced availability of sugary food and drink, and reduced promotion through media and advertising. HROH coverage will be important to ensure access to care. Third, community action is a powerful resource; thus, groups such as parents should be facilitated to advocate for health. Communities should therefore work together to strengthen oral health education and promotion measures by advocating affirmative health behaviours together with promoting prevention and uptake of dental care. Fourth, we suggest *health-promoting schools* as a means to promote oral health, develop personal skills and deliver basic preventive measures to children across both schools and kindergartens [44]; this is additionally a setting where simple dental care may be delivered. Fifth, dental professionals, supported by government, should be required to embrace evidence-based prevention and thus focus on delivering high quality contemporary care involving prevention rather than merely reparative treatment of disease.

Based on the fact that dental care required within the child population involves procedures within the scope of practice of dental hygienists, we strongly recommend their development in China as currently only two categories of workforce, namely dentists and assistant dentists, exist. Dental hygienists can carry out all preventive care, as well as scaling and polishing, are shown to have a positive effect on patients offering preventive care and outreach services [45], and regarded as making great contributions to improving oral health through school-based initiatives [46]. They can work at schools/kindergartens or in communities, or move from one place to another during their work, to provide these basic preventive measures for children and community residents, and refer to dentists or assistant dentists if restorations or other more complicated measures are required. A further consideration would be the development of dental therapists who can additionally provide routine fillings [34, 39, 40, 47]. In either case, dentists and assistant dentists would be able to apply their expertise to more complex care. As dental hygienists do not require as long and as expensive an education, it may be much easier to employ them in the first instance to fill in the huge imbalance between current and actual HROH and address the growing prevention needs in China. In support of this, students during their dental education should have experience of working in areas of high need, to attract dental professionals into these areas voluntarily. Although it seems the most reasonable option for Chinese HROH in future from our findings, we recognise there is still a long way to go to change current policies and public acceptance. Further research, including feasibility and pilot studies, should investigate the socio-economic effects and cost-effectiveness of developing HROH skill mix in China and ensure appropriate funding models.

Some limitations of this research need to be addressed. First, we used data from the 3^rd^ national Oral Health Survey in 2005; however, this remains the contemporary reference of oral health in China [11, 29, 30]. For sample weighting, we were using a contemporary nationwide census (the 5th National Demographic Census in China) in line with the oral health survey, and the workforce data are also estimated based on this census. Although the 12-year-old population reduced by 37.3% in the 6th Census in 2010 owing to the very strict one-child policy during 1990s, the total population of China is still growing by 6.8% [31, 48], and ageing. Following transition to a two-child policy in 2015 [49], the population of school-age children is predicted to rebound in future [50]. Second, our study sample included 87% of the participants with questionnaire responses, which may raise some concerns about the impact of missing data and the representativeness of the sample. However, the major difference between the study sample and those excluded because of missing values was that more caries free participants were included in the analysis, suggesting our estimated workforce needs are likely to be conservative. Third, we adopted our own methods for caries risk assessment based on contemporary evidence. Though this has not been used in previous studies, we recommend this method for our present study as we have limited information from the national survey (e.g. questions on income level were not included in the questionnaire for 12-year-olds). We also employed maximum and minimum professional intervention models to obtain a range of timings and workforce requirements based on philosophical levels of intervention, which we hope, because of its transparency, will facilitate debate over HROH requirements. Focusing on the minimum intervention model as more realistic allows for some to attend more regularly and others less. Fourth, our formulae used in estimation of the needs have adopted some reference data (i.e. timings for each item from a consulting group, proportion of working hours on 12-year-olds from the UK literature) as there are no corresponding published data from China. The members of the consulting group are all from Peking University School and Hospital of Stomatology, an institution in the top three positions in dentistry in China, dentists working there may need less operating time given their expertise and training. This further suggests that our estimation of workforce needs, albeit large, may still represent something of an underestimate. In addition, the differences between China and England are important to consider when extrapolating the findings, as there are differing population age structures. In China, 12-year-olds accounted for 1.98% of the whole population in 2000, and 1.16% in 2010 [31, 48]; whereas in England, the corresponding percentage in 2015 was 1.08% [51], and relates to Wanyonyi et al.’s analysis of workforce needs and skill mix [34]. The estimate of workforce capacity used in treating 12-year-old patients used in this study was 1.27% [34], which is slightly higher than national proportion in England, but still between the two corresponding percentages of China in 2000 and 2010 [31, 48]. Thus, we consider this proportion (1.27%) is a fair representative estimate when estimating workforce requirements for the whole population. The extent to which 12-year-old oral health reflects population oral health needs and HROH needs across different populations should be explored in future research; however, the implications for care will depend on healthcare philosophy as well as need. Fifth, as already explored, our models used the results of 12-year-olds to extrapolate the estimation of the whole population. At 12 years of age, children are at the start of their permanent dentition, so it is good choice to consider this age group and investigate their needs for dental care initially. However, as the prevalence and severity of common dental diseases (i.e. dental caries and the ramifications of treatment and periodontal disease) increase with age [52, 53], we argue that the actual needs of dental care for the whole population may be higher than anticipated by this model. Sixth, we assumed that every erupted posterior tooth needs to be sealed, regardless of its morphology. However, sealants are effective in caries prevention [54], and it is routinely recommended to seal the permanent teeth in those children with higher risk of caries [15, 55]. Seventh, there are certain shortcomings of needs-led approach [24], including the absence of economic analysis; however, we suggest that our transparent model is an important starting point for discussion and future research, particularly as it places population health needs and health outcomes at the centre of HROH considerations.

## Conclusions

Our analysis of national survey data using a needs-led approach based on risk assessment in 12-year-old sample shows that there is a large gap between dental workforce needs and actual workforce numbers in China. Governments need to make provision for rural areas in particular; we recommend that the skill mix of the dental team should be expanded and that contemporary clinical and preventive care are delivered in community settings where possible.

## Additional file

Additional file 1: Table S1. Timings for dental treatment and prevention measures in the UK and China. (DOCX 31 kb)

## Abbreviations

AAPD: American Academy of Paediatric Dentistry
ADA: American Dental Association
CPI: Community periodontal index
DMFT: Number of decayed, missing and filled permanent teeth
FDI: World Dental Federation
GDP: Gross domestic product
HRH: Human resources for health
HROH: Human resources for oral health
IBM: International Business Machine
maxPIM: Maximum professional intervention model
minPIM: Minimum professional intervention model
NICE: UK National Institute for Health and Care Excellence
NY: New York
PHE: Public Health England
SPSS: Statistical Product and Service Solutions
UK: United Kingdom
WHO: World Health Organization
