# Supplementary material for: Meeting the oral health needs of 12-year-olds in China: human resources for oral health
Source: BMC Public Health. 2017 Jun 20;17:586. doi: 10.1186/s12889-017-4384-7 (PMC5477685; doi:10.1186/s12889-017-4384-7)
Supplement: Additional file 1: Table S1. — Timings for dental treatment and prevention measures in the UK and China. (DOCX 31 kb) [file 12889_2017_4384_MOESM1_ESM.docx]

Additional file Table S1 for

**Meeting the Oral Health Needs of 12-year-olds in China: Human Resources for Oral Health**

**Xiangyu Sun^1,2^, Eduardo Bernabé^2^, Xuenan Liu^1^, Shuguo Zheng^1,*^, Jennifer E. Gallagher^2,^**^*^

^1^Department of Preventive Dentistry, Peking University School and Hospital of Stomatology, National Engineering Laboratory for Digital and Material Technology of Stomatology, Beijing Key Laboratory of Digital Stomatology, Haidian District, Beijing, People’s Republic of China

^2^King’s College London Dental Institute at Guy’s, King’s College and St Thomas’ Hospitals, Population and Patient Health Division, London, United Kingdom

***Corresponding authors.**

**Professor Jennifer E. Gallagher MBE** (jenny.gallagher@kcl.ac.uk)

King’s College London Dental Institute at Guy’s, King’s College and St Thomas’ Hospitals, Population and Patient Health Division, Denmark Hill Campus, Bessemer Road, London SE5 9RS, United Kingdom.

**Professor Shuguo Zheng** (zhengsg86@gmail.com)

Department of Preventive Dentistry, Peking University School and Hospital of Stomatology, National Engineering Laboratory for Digital and Material Technology of Stomatology, Beijing Key Laboratory of Digital Stomatology, 22 Zhongguancun Avenue South, Beijing 100081, People’s Republic of China.

**E-mail addresses of all authors**: Xiangyu Sun (allon627@163.com), Eduardo Bernabé (eduardo.bernabe@kcl.ac.uk), Xuenan Liu (lxn1968@163.com), Shuguo Zheng (zhengsg86@gmail.com), Jennifer E. Gallagher (jenny.gallagher@kcl.ac.uk)

**Table S1.** Timings for dental treatment and prevention measures in the UK and China

| Mean timings needed  (in minutes) | BDA Heathrow  1999 | Wanyonyi et al.  2015 | China (PKUSS)  2016 |
| --- | --- | --- | --- |
| Tooth restorations | 18.2^a^ | 17.6^a^ | 21.7^b^ |
| Regular check-ups | 11.3^a^ | 11.3^a^ | 16.3^a^ |
| Fluoride varnish application | - | 5.0^a^ | 7.6^a^ |
| Fissure sealants | 9.9^a^ | 18.2^a^ | 6.9^b^ |
| Scaling and polishing | 15.1^a^ | 15.1^a^ | 31.1^a^ |

^a^ Timings for each time.

^b^ Timings per tooth.
